# A comparative study on bath and horn ultrasound‐assisted modification of bentonite and their effects on the bleaching efficiency of soybean and sunflower oil: Machine learning as a new approach for mathematical modeling

**DOI:** 10.1002/fsn3.4300

**Published:** 2024-07-09

**Authors:** Elahe Abedi, Mehran Sayadi, Maryam Mousavifard, Farzad Roshanzamir

**Affiliations:** ^1^ Department of Food Science and Technology, Faculty of Agriculture Fasa University Fasa Iran; ^2^ Department of Food Safety and Hygiene, School of Health Fasa University of Medical Sciences Fasa Iran; ^3^ Department of Civil Engineering, Faculty of Engineering Fasa University Fasa Iran

**Keywords:** machine learning, ultrasound‐assisted bleaching, ultrasound‐treated bleaching clay, XGBoost model

## Abstract

In this study, the effect of high‐power bath and horn ultrasound at different powers on specific surface area (*S*_BET_), total pore volume (*V*_total_), and average pore volume (*D*_ave_) of bleaching clay was examined. After subjecting the bleaching clay to ultrasonication treatment, the SBET values demonstrated an escalation from 31.4 ± 2.7 m^2^ g^−1^ to 59.8 ± 3.1 m^2^ g^−1^ for HU200BC, 143.8 ± 3.9 m^2^ g^−1^ for HU400BC, 54.4 ± 3.6 m^2^ g^−1^ for BU400BC, and 137.5 ± 2.8 m^2^ g^−1^ for BU800BC. The mean pore diameter (*D*_ave_) declined from 29.7 ± 0.14 nm in bleaching clay to 11.3 ± 0.13 nm in HU200BC, 8.3 ± 0.12 nm in HU400BC, 16.7 ± 0.14 nm in BU400BC, and 9.6 ± 0.12 nm in BU800BC. Therefore, horn ultrasound‐treated bleaching clay significantly increased *S*_BET_ and *V*_total_, indicating improved adsorption capacity. Moreover, to establish the relationship between bleaching parameters, seven multi‐output ML regression models of Feedforward Neural Network (FNN), Random Forest (RF), Support Vector Regression (SVR), Multi‐Task Lasso, Ridge regression, Extreme Gradient Boosting (XGBoost), and Gradient Boosting are used, and compared with response surface methodology (RSM). ML has revolutionized the understanding of complex relationships between ultrasonic parameters, oil color, and pigment degradation, providing insights into how various factors such as temperature, ultrasonic power, and time can influence the bleaching process, ultimately enhancing the efficiency and precision of the treatment. The XGBoost model showed outstanding performance in predicting the target variables with a high *R* ^2^‐train up to 1, *R* ^2^‐test up to .983, and a minimum mean absolute error (MAE) of 0.498. The lower error between the predicted and experimental values implies the superiority of the XGBoost model to predict outcomes rather than RSM. It represents the suitability of bath ultrasound as a mild condition for low‐pigmented oil bleaching. Finally, the Bayesian optimization method in conjunction with XGBoost was used to optimize the amount of bleaching clay and energy consumption, and its performance was compared with RSM. It was observed that the consumption of bleaching clay was reduced by approximately 60% for sunflower oil and 30%–35% for soybean oil.

## Graphical abstract

Most of the studies conducted so far have relied on experimental methods or empirical modeling techniques. Experimental methods can be time‐consuming, expensive, and complex, requiring sophisticated and costly experimental facilities. Additionally, conducting a series of experiments on a wide range of food products can be impractical. To overcome these challenges, ML‐based predictive approaches have been introduced in food processing. One such approach is the use of Artificial Neural Networks (ANN), which is a powerful tool for biological predictive modeling. ANN models excel at handling problems involving massive combinatorial spaces or nonlinear processes that are not easily solvable using traditional approaches. ML approaches, such as ANN, are increasingly being considered by food engineers and scientists due to their non‐destructive nature, simplicity, and real‐time monitoring capabilities. Nemours reviews declared that ML approaches offer superior tools for modeling complex, dynamic, highly nonlinear, and ill‐defined scientific and engineering problems in food processing and food safety. Researchers have attempted to use ML‐based approaches in various areas of food processing, including energy and exergy prediction, predicting physicochemical properties, and mathematical modeling. ML techniques offer great potential for improving the efficiency and effectiveness of ultrasound‐assisted bleaching processes. The aim of this study is to introduce a practical model and optimization approach for ultrasound‐assisted bleaching of soybean and sunflower oil using the ML technique. To the best of our knowledge, no previous study has reported this specific approach. In this study, two ultrasonic modes (horn and bath) with various experimental conditions and two types of oil with different color and pigment characteristics were mathematical modeled using the multi‐output ML technique, allowing for practical operation conditions with moderate to severe ultrasonic exposure to aim bleaching clay reduction.

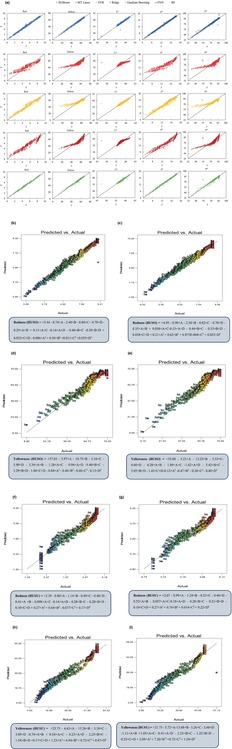

## 1 INTRODUCTION

In the commercial process of edible oil refining, bleaching is employed to decrease the presence of pigments like chlorophyll and carotenoids. Additionally, it eliminates undesired minor components such as metals, phospholipids, and oxidation products (Silva et al., 2014). The mechanism of bleaching in edible oil refining can involve adsorption, heating, or chemical oxidation. When using clays or earths for bleaching, it primarily functions as an adsorption process. This adsorption process involves complex interactions between the clay particles and undesired molecules. These interactions can include physical mechanisms like the molecular sieving effect, where undesired compounds are trapped inside the pores of the clays. Additionally, the attraction effect of Van der Waals forces and, in some cases, chemical reactions via ionic bonds can also occur during the bleaching process (Aachary et al., 2016; Abedi et al., 2021).

Several alternative techniques have been developed to enhance bleaching performance and overcome the limitations of conventional methods. These include membrane technology, supercritical fluid extraction, and, more recently, ultrasound‐assisted methods. Ultrasound‐assisted bleaching, in particular, offers several advantages over traditional bleaching procedures and is gaining interest in commercial oil refining. Ultrasound‐assisted bleaching utilizes ultrasonic irradiation to improve the adsorption process through various mechano‐chemical routes. However, it is important to note that the improved bleaching observed after sonication is mainly attributed to physical effects rather than chemical alterations. This technique is believed to enhance the efficiency of adsorption and pigment removal, leading to improved oil quality (Abedi et al., 2020, 2021; Asgari et al., 2017; Icyer & Durak, 2018; Su et al., 2013).

High‐intensity ultrasonic waves have been found to significantly improve the conventional bleaching process of edible oils (Jahouach‐Rabai et al., 2008). When ultrasonic waves propagate through the bleaching chamber, they generate cavitation. The efficient bleaching observed after the application of ultrasound is primarily associated with an improved physical adsorption mechanism (Asgari et al., 2018; Roohi et al., 2019). The mechanical effects of high‐intensity ultrasonic waves, particularly the sudden, intense implosion of cavitation bubbles, induce fluid turbulence and enhance dynamic interactions between the adsorbent and adsorbate. This leads to increased mass transfer and improved adsorption efficiency. Additionally, microstreaming and microjets at the solid–liquid boundaries caused by ultrasonic cavitation result in erosion of the adsorbent surface, creating more adsorptive sites. The use of ultrasound‐assisted bleaching offers several notable benefits, including enhanced bleaching efficiency, reduced bleaching clay usage, shorter process time, and, ultimately, a decrease in bleaching costs and losses.

Several researchers have explored the application of sonication technology for bleaching olive (Asgari et al., 2017, 2018; Jahouach‐Rabai et al., 2008), soybean (Abedi et al., 2015, 2017, 2020, 2021; Roohi et al., 2019), hempseed oil (Aachary et al., 2016), and canola or rape seed oils (Icyer & Durak, 2018; Su et al., 2013). By exploring ultrasound‐assisted bleaching, researchers aim to develop more sustainable, efficient, and environmentally friendly food processing approaches for removing color pigments and other impurities in edible oil refining, which can help mitigate the drawbacks associated with conventional bleaching methods.

Over the past few decades, significant advancements have been made by world food researchers in obtaining important information and developing bleaching technology. However, most of the studies conducted so far have relied on experimental methods or empirical modeling techniques. Experimental methods can be time‐consuming, expensive, and complex, requiring sophisticated and costly experimental facilities. Additionally, conducting a series of experiments on a wide range of food products can be impractical. To overcome these challenges, ML‐based predictive approaches have been introduced in food processing. One such approach is the use of Artificial Neural Networks (ANN), which is a powerful tool for biological predictive modeling. ANN models can be well‐suited for handling certain types of problems involving complex, nonlinear relationships or large combinatorial spaces, where they may outperform traditional approaches in some cases (Goodfellow et al., 2016; Schmidhuber, 2015; Shalev‐Shwartz & Ben‐David, 2014). ML approaches, such as ANN, are increasingly being considered by food engineers and scientists due to their non‐destructive nature, simplicity, and real‐time monitoring capabilities. Nemours reviews declared that ML approaches offer superior tools for modeling complex, dynamic, highly nonlinear, and ill‐defined scientific and engineering problems in food processing and food safety. Researchers have attempted to use ML‐based approaches in various areas of food processing, including energy and exergy prediction, predicting physicochemical properties, and mathematical modeling (Deng et al., 2021; Khan et al., 2022; Saha & Manickavasagan, 2021; Wang et al., 2022). Alongside ANNs, several other machine learning algorithms have also been explored in the field of food science and technology. The eXtreme Gradient Boosting (XGBoost) algorithm has been successfully employed in soybean yield prediction, outperforming other county‐level models such as linear regression, random forest, k‐nearest neighbor, ANN, support vector regression, long short‐term memory, and deep neural networks (Li et al., 2023) Furthermore, the XGBoost algorithm has been utilized to develop highly accurate classification models for identifying differently ripened bananas based on their hyperspectral images, accurately differentiating between naturally ripened and artificially ripened samples (He et al., 2022). Additionally, Wang et al. (2021) have demonstrated the reliability of the XGBoost model in screening and predicting food‐derived antihypertensive peptides with high throughput and accuracy, further verifying their findings through peptide–protein docking analysis.

Support vector regression (SVR) has also proven to be a valuable tool in food science research. Sim et al. (2023) employed SVR with a radial basis function kernel to successfully predict an integrated dataset of stable isotope ratios and multi‐element composition of specialty green coffee beans from different geographical origins using near‐infrared hyperspectral imaging data, corroborating the findings of Sahraei et al. (2021), who compared ANN and SVR for predicting stable isotope ratios of water and found SVR to provide the best predictions.

The random forest (RF) algorithm has been explored for adulteration detection in food products. de Santana et al. (2019) used RF, combined with the artificial generation of outliers from authentic samples, as a novel approach for detecting adulteration in evening primrose oils and ground nutmeg, demonstrating that the RF‐based method outperformed or matched the performance of other commonly used techniques like PLS‐DA and SIMCA. Jeong et al. (2016) further showcased the effectiveness of the RF machine learning method in predicting crop yields of wheat, maize, and potatoes at global and regional scales, outperforming multiple linear regression models.

The least absolute shrinkage and selection operator (LASSO) algorithm has been utilized to examine the association between dietary intakes, risk factors, and self‐reported breast cancer in women aged 50 and above using data from the National Health and Nutrition Examination Survey (NHANES) (McEligot et al., 2020). Additionally, He et al. (2024) employed least angle regression (LARS) combined with LASSO to select the most important wavelengths from hyperspectral imaging data, which were then used to develop accurate partial least squares (PLS) regression models for rapid quantification of vitamin C and reducing sugar levels in sweet potato roots. Gradient‐boosting machine (GBM) models have also been explored in food science research. Golden et al. (2019) used GBM models, in addition to random forest (RF) models, to describe and predict the prevalence of Listeria spp. in soil and feces samples from pastured poultry farms based on meteorological factors, demonstrating the superior performance of GBM models compared to RF models, particularly for predicting Listeria spp. prevalence in soil. Similarly, Branstad‐Spates et al. (2023) developed a predictive model using the gradient boosting algorithm to predict aflatoxin contamination in corn in Iowa, USA, utilizing historical data on corn contamination, meteorological factors, satellite imagery, and soil properties. Ridge regression, a technique to handle multicollinearity, has been employed by Devi et al. (2023) to determine the major factors influencing the agricultural production of wheat, gram, paddy, and cotton in the Haryana state of India, demonstrating the appropriateness of ridge regression in the presence of multicollinearity compared to ordinary least squares. Furthermore, Kutty et al. (2020) proposed a weighting approach using ridge penalization‐based regression to address the challenge of high correlation among environmental aspects in the assessment of eco‐efficiency indicators and validated the performance of this approach using economic and environmental footprint data from 20 food industries in the United States. These examples highlight the versatility and effectiveness of various machine learning algorithms.

ML techniques offer great potential for improving the efficiency and effectiveness of ultrasound‐assisted bleaching processes. This study aims to introduce a practical model and optimization approach for ultrasound‐assisted bleaching of soybean and sunflower oil using the ML technique. To the best of our knowledge, no previous study has reported this specific approach. In this study, two ultrasonic modes (horn and bath) with various experimental conditions and two types of oil with different color and pigment characteristics were mathematical modeled using the multi‐output ML technique, allowing for practical operation conditions with moderate to severe ultrasonic exposure to aim bleaching clay reduction.

## 2 MATERIALS AND METHODS

### 2.1 Materials

The bleaching process utilized neutralized soybean and sunflower oil, which was obtained from Narges Oil Company located in Shiraz, Fars Province, Iran. All the chemicals required for this study were purchased from Merck Co. Germany and were of analytical grade.

### 2.2 Ultrasound equipment

#### 2.2.1 Ultrasonic bath

For UAB, an ultrasonic cleaning bath from Pacisa SA in Spain was utilized. The bath operates at 25 kHz and has a working power of 1000 W. The ultrasonic processor is a rectangular chamber measuring 30 cm × 15 cm × 15 cm and is equipped with a temperature control unit. The bath UAB of oils was conducted at two amplitudes (20% and 80% W).

#### 2.2.2 Ultrasonic horn

The oil samples (50 mL) were subjected to sonication using an ultrasonic generator of the UP400S Hielscher type. The generator had a power output of 400 W and operated at a frequency of 24 kHz. An immersible probe was used, which was positioned in a 100‐mL cylindrical jacket glass vessel. The probe was immersed in the liquid at the top of the vessel, transmitting sound vibrations into the oil sample through a titanium alloy rod with a diameter of 14 mm. The probe ultrasound‐assisted bleaching of oils was conducted at two amplitudes (50% and 100%).

### 2.3 Bleaching process

During the initial pretreatment, it was observed that the amount of primary color in soybean oil was higher compared to sunflower oil. As a result, a higher amount of color‐removing clay was required for soybean oil compared to sunflower oil. The bleaching process involved conducting experiments with activated bentonite clay added to a 50‐mL oil sample at different concentrations (0.5%, 1%, 1.5%, and 2% w/v) for soybean oil and (0.5%, 1%, and 1.5% w/v) for sunflower oil. Before the ultrasonic process, acid‐activated bentonite by ultrasounds by bath and horn was added to the 50 mL oil sample in all experiments.

The ultrasonic bleaching process was carried out using a horn ultrasonic processor from UP400S Hielscher (400 W, 24 kHz) and an ultrasonic cleaning bath from Pacisa SA (1000 W, 25 kHz). The influence of different bleaching treatments was evaluated by varying the levels of ultrasonic power setting (0%, 50%, 100%; horn ultrasound and 0, 20%, and 80%; bath ultrasound), bleaching clay dosage (0%–2% for soybean and 0%–1.5% for sunflower oil), process temperature (35, 45, 55, and 65°C), and time (5, 10, 15, 20, 25, 30 min). The Lovibond red color was chosen as it is a commonly used preliminary quality criterion. According to American trading rules, a maximum of 2.5 red is the final acceptable color for bleached oils (Rossell, 1991), and so reaching this value was the aim of this study. After agitation, the mixture was filtered through a centrifuge at 14,000 rpm for 20 min, and the resulting supernatant solution was further filtered using the Whatman 42 filter paper. The bleaching process, conducted without the use of ultrasonics, served as the control. All tests were performed in triplicate.

### 2.4 Color measurement

The color of the oil sample was determined using two different instruments. First, a Lovibond 5¼ Tintometer model F was employed. This instrument uses a glass cell with an optical path length of 10 mm, and the colors of the oil sample were compared with the CIE L*, a*, b* color systems. The Colorflex instrument from Reston, VA, USA, was used to measure the color of the oil sample in the Hunter Lab system. A 20‐mm tube was used as the sample cell for this measurement (Abedi et al., 2015).

### 2.5 Determination of chlorophyll and carotenoid

To determine the chlorophyll and carotenoid contents of the bleached oil sample, the method described by Abedi et al. (2015) was employed. In this method, 7.5 g of oil were dissolved in cyclohexane to reach a final volume of 25 mL. The concentration of chlorophyll and carotenoid in the oil sample was then measured spectrophotometrically using a UV–VIS spectrophotometer (UV S‐2100; Scinco, Seoul, South Korea) at wavelengths of 670 nm and 470 nm, respectively. The following Equations (1) and (2) were used for the measurement:

(1) $$\text{Chlorophyll}\;\left(\mathrm{\mu g}/\mathrm{kg}\right)=A_{670}\times 10^{9}/613\times 100\times d$$

(2) $$\text{Carotenoid}\;\left(\mathrm{mg}/\mathrm{kg}\right)=A_{470}\times 10^{6}/2000\times 100\times d$$

where *A*: absorbance; *d*: spectrophotometric cell diameter (1 cm).

### 2.6 Bleaching clay characterization

The Brunauer–Emmett–Teller specific surface area (SBET), pore volume, and average pore size of the natural, horn‐ultrasonicated bleaching clay (200 and 400 W) and bath‐ultrasonicated bleaching clay (400 and 800 W) were determined using low‐temperature nitrogen physisorption at 77 K. This measurement was performed within the vapor pressure range (P/P0) of 0.01–0.99 using a Quantachrome instrument from the USA. Before the physisorption measurement, the samples were subjected to degassing at a temperature of 150°C for a duration of 8 h. This process removes any adsorbed gases or moisture from the sample, ensuring accurate and reliable measurement of the surface area and pore characteristics.

### 2.7 Mathematical modeling by machine learning

One crucial step in the utilization of ML models is preprocessing data, as it can significantly impact the performance and accuracy of models. Data normalization is an important aspect of preprocessing that brings all features to a similar scale. In this study, the Min–Max Scaler method is used to transform the values of the features so that they are mapped to a given range, typically between 0 and 1.

In this method of scaling, the minimum and maximum values of each feature are calculated from the original dataset. These values represent the range of the feature values. To scale each feature, the Min–Max Scaler uses Equation (3):

(3) $$X\;\text{scaled}=\frac{x-x_{\min}}{x_{\max}-x_{\min}}$$

This formula transforms each feature value (*x*) to a scaled value (*x*_scaled_) within the desired range. The scaling process is applied individually to each feature, subtracting the minimum value and dividing the result by the range (maximum value minus minimum value). This scaling technique is particularly useful when features have different scales and ranges. It helps to normalize the data and prevent certain features from dominating the model's learning process. Seven specific regression models were selected for analysis: FNN, RF, SVR, MT Lasso, Ridge, XGBoost, and GB. The hyperparameters of each model were initially fine‐tuned using Grid Search to optimize their performance. The best model with optimized hyperparameters was then utilized to predict the experimental data. Due to the significant impact of hyperparameters on predictive performance, it is crucial to carefully tune them. This study focused on adjusting the number of hidden layers, number of neurons per hidden layer, and type of activation function for FNN; number of trees and maximum depth of trees for RF; type of Kernel function, regularization parameter, and Epsilon for SVR; regularization parameter for MT Lasso; regularization parameter for ridge; number of trees, maximum depth of trees, and learning rate for XGBoost and GB.

#### 2.7.1 Multi‐output regression

The ultrasound‐assisted bleaching cases involve four input variables: the percent of clay, power, time in minutes, and temperature, and the control experiments include three input variables: the percent of clay, time (min), and temperature. In contrast, in both cases, there are five output variables, including red, yellow, L*, a*, and b*.

Multi‐output regression models were employed to tackle the challenge of predicting and evaluating the results with multiple output variables. As it was mentioned previously, the chosen models include seven methods of feedforward neural networks (FNN), random forest, support vector regression (SVR), multi‐task lasso, ridge regression, XGBoost, and gradient boosting, which are listed in Table 1. While some of the models used in this study, such as feedforward neural networks, random forests, and gradient boosting, inherently support multi‐output regression, others, like support vector regression (SVR), multi‐task lasso, and ridge regression, are not specifically designed for multi‐output regression. However, by applying joint modeling techniques (MultiOutputRegressor in Python), these models can be adapted to handle multiple outputs effectively (Fieuws & Verbeke, 2006). In joint modeling for multi‐output regression models, the strategy involves simultaneously modeling all output variables with shared latent factors to capture dependencies and correlations among the outputs, allowing for more accurate predictions and improved interpretability of the relationships between the variables (Borchani et al., 2015). It allows for the exploitation of correlations between the variables, leading to more accurate predictions. By considering the joint distribution of the output variables, the models can better understand and capture the interactions between red, yellow, L*, a*, and b*, resulting in improved prediction accuracy. The details of each model are described in a Appendix S1.

**TABLE 1:** Different machine learning regression models.

Regression models	Equations	Parameters	References
Feed‐forward Neural Network (FNN) with two hidden layers	*Y* = *f*(*W*_2_ × *f*(*W*_1_ × *X* + *b*_1_) + *b*_2_)	*Y*: the predicted output or target variable, *X*: the input features or independent variables; *W*_1_ and *W*_2_: the weights of the neural network's hidden and output layers, respectively; the biases *b*_1_ and *b*_2_ are associated with the hidden and output layers, respectively; *F*: the activation function	Golab et al. (2022), Gülcü (2022), Omid et al. (2009)
Random Forest	*Y* = Σ (*h*_*i*_(*X*) × *w*_*i*_)	*w*_*i*_: the weight or importance assigned to each decision tree's prediction	Rajković et al. (2023), Sharma et al. (2023)
Support Vector Regression (SVR)	*Y* = Σ (*α*_*i*_ × *K*(*X*_*i*_, *X*)) + *b*	*α*_*i*_: the Lagrange multipliers associated with the support vectors; *K*(*X*_*i*_, *X*): the kernel function applied to the support vectors and the input features; *b*: the bias term	Huang et al. (2022), Zhang and O'Donnell (2020), Zhong et al. (2013)
Multi‐Task Lasso	1/(2 × *n*) × ||*Y* − *XW*||^2^_*F*_ + *α* × ||*W*||_21_	*W*: the matrix of coefficients || ||_*F*_: the Frobenius norm *α*: the regularization parameter	Lozano and Swirszcz (2012), Thung and Wee (2018), Zhou et al. (2010)
Ridge regression	*Y* = *β*_0_ + *β*_1_ × *X*_1_ + *β*_2_ × *X*_2_ + … + *β*_*n*_ × *X*_*n*_ + *λ* × Σ(*β*_*i*_^2^)	*β*_0_: the bias term *β*_1_, *β*_2_, …, *β*_*n*_: the weights of the input features *Λ*: the regularization parameter Σ(*β*_*i*_^2^): the penalty term	Alaoui and Mahoney (2015), Hastie (2020), Rajan (2022)
Extreme Gradient Boosting (XGBoost)	*Y* = Σ (*w*_*i*_ × *h*_*i*_(*x*)) + *b*	*W*_*i*_: the weight or importance of the *i*th weak learner (decision tree) *h*_*i*_(*x*): the prediction of the *i*th weak learner on the input data *x*	Chen et al. (2015), Huber et al. (2022), Wang and Ross (2018)
Gradient Boosting	*Y* = Σ (*η* × fi(*x*))	*Η*: the learning rate, which controls the contribution of each weak learner fi(*x*): the prediction of the *i*th weak learner (decision tree) on the input data *x*	Costa et al. (2019), Konstantinov and Utkin (2021), Sipper and Moore (2022)

*Note*: *Y*: The predicted output or target variable; *X*: the input features or independent variables.

#### 2.7.2 Evaluation factors

1. *R* ^2^ is a statistical measure that represents the proportion of the variance in the dependent variable (target variable) that can be explained by the independent variables in a regression model. It ranges from 0 to 1, where 1 indicates a perfect fit of the model to the data. *R* ^2^ helps assess how well the model fits the data and how well it can predict future observations. Higher *R* ^2^ values indicate a better fit and more accurate predictions, while lower values indicate a poorer fit.

(4) $$R^{2}=1-\left(\mathrm{SSR}/\mathrm{SST}\right)$$

where SSR (sum of squared residuals) is the sum of the squared differences between the predicted and actual values: SSR = Σ(*y*_pred_ − *y*_actual_)^2^. SST (Total Sum of Squares) is the sum of the squared differences between the actual values and the mean of the actual values: SST = Σ(*y*_actual_ − $\bar{y}$_actual_)^2^.

2 Mean absolute error (MAE) is a metric that measures the average absolute difference between the predicted and actual values in a regression model. It provides a measure of the average magnitude of errors without considering their direction. MAE is calculated by taking the average of the absolute differences between predicted and actual values. MAE is useful for evaluating the overall accuracy of a model, and a lower MAE indicates better prediction performance.

(5) $$\mathrm{MAE}=\left(1/n\right)\times \Sigma |y_{\text{pred}}–y_{\text{actual}}|$$

where *n* is the number of data points and |*y*_pred_ – *y*_actual_| represents the absolute difference between the predicted and actual values.

3 Mean squared error (MSE) is another metric used to measure the average squared difference between the predicted and actual values in a regression model. It penalizes larger errors more than MAE because of the squaring operation. MSE is calculated by taking the average of the squared differences between predicted and actual values. MSE is commonly used in regression models as it provides a measure of the average magnitude of errors and is useful for comparing the performance of different models. Like MAE, a lower MSE indicates better prediction performance.

(6) $$\mathrm{MSE}=\left(1/n\right)\times \Sigma {\left(y_{\text{pred}}–y_{\text{actual}}\right)}^{2}$$

where *n* is the number of data points and (*y*_pred_ – *y*_actual_)^2^ represents the squared difference between the predicted and actual values.

#### 2.7.3 Correlation

The correlation between the input parameters (clay, power, time, and temperature) and the output parameters (red, yellow, L*, a*, and b*) was demonstrated using the Pearson correlation method (Equation 7). The correlation coefficient ranges from −1 to 1, where values closer to −1 or 1 indicate a stronger correlation, and values closer to 0 indicate a weaker correlation.

(7) $$r=\frac{\sum \left(x_{i}-\bar{X}\right)\left(y_{i}-\bar{Y}\right)}{\sqrt{\sum {\left(x_{i}-\bar{x}\right)}^{2}{\left(y_{i}-\bar{y}\right)}^{2}}}$$

In this equation, *x*_*i*_ and *y*_*i*_ represent the individual data points of the inputs and outputs, respectively. $\bar{X}$ and $\bar{Y}$ represent the means (or averages) of the input and output variables.

### 2.8 Response surface methodology (RSM)

In this study, the experimental design was conducted using Design Expert (version 6.0.5). These software packages were utilized for regression analysis to obtain the regression coefficients from the experimental data (Myers et al., 2004). To investigate the impact of various factors on the bleaching efficiency of soybean and sunflower oil, a general factorial design was employed. This design considers factors with different numbers of levels and enables the creation of an experiment encompassing all possible combinations of the factor levels namely, ultrasonic power, bleaching clay, temperature, and time. The experimental data were fitted using Equation (8), which represents a second‐order polynomial equation. This equation includes the linear and interaction effects of each factor.

(8) $$Y=\beta_{0}+\sum_{i=1}^{k}\beta_{i}X_{i}+\sum_{i=1}^{k}\beta_{\mathrm{ii}}X_{i}^{2}+\sum_{i=1}^{k-1}\sum_{j=2}^{k}\beta_{\mathrm{ij}}X_{i}X_{j}$$

In Equation (8), *Y* represents the predicted response variable. *X*_*i*_ and *X*_*j*_ represent the independent factors being studied. The equation includes various terms such as the offset term (*β*_0_), linear coefficients (*β*_*i*_) for each factor, quadratic coefficients (*β*_*ii*_) for each factor, and interaction coefficients (*β*_*ij*_) for the interactions between factors.

## 3 RESULTS AND DISCUSSION

### 3.1 Characterization of the bleaching clay

The SBET and pore structure of the BC, HU200BC, HU400BC, BU400BC, and BU800BC were examined and presented in Table 2. After being treated with horn and bath ultrasound at different powers, the size of the bleaching clay was significantly reduced. The surface morphology was significantly altered and exhibited a textured surface characterized by curls and layers. Following the treatment of the BC through ultrasonication, the S_BET_ values exhibited an increase from (31.4 ± 2.7 m^2^g^−1^) to the HU200BC (59.8 ± 3.1 m^2^g^−1^), HU400BC (143.8 ± 3.9 m^2^g^−1^), BU400BC (54.4 ± 3.6 m^2^g^−1^), and BU800BC (137.5 ± 2.8 m^2^g^−1^). The average pore diameter (*D*_ave_) decreased from 29.7 ± 0.14 nm (BC) to 11.3 ± 0.13 nm (HU200BC), 8.3 ± 0.12 nm (HU400BC), 16.7 ± 0.14 nm (BU400BC), and 9.6 ± 0.12 nm (BU800BC). Ultrasonication has the potential to create pore spaces within the bleaching clay, and the probe presented a direct influence on creating pore space compared to bath ultrasonic.

**TABLE 2:** Specific surface area (*S*_BET_), total pore volume (*V*_total_), and average pore volume (*D*_ave_) of native and sonicated bleaching clay samples.

	*S*_BET_ (m^2^ g^−1^)	*V*_total_ (cm^3^ g^−1^)	*D*_ave_ (nm)
BC	31.4 ± 2.7	0.11 ± 0.03	29.7 ± 0.14
HU200BC	59.8 ± 3.1	0.19 ± 0.02	11.3 ± 0.13
HU400BC	143.8 ± 3.9	0.37 ± 0.03	8.3 ± 0.12
BU400BC	54.4 ± 3.6	0.16 ± 0.02	16.7 ± 0.14
BU800BC	137.5 ± 2.8	0.28 ± 0.04	9.6 ± 0.12

### 3.2 Predicting UAB using machine learning regression models

Table 3 displays the outcomes of the ML regression models utilized for predicting the levels of UAB via bath and horn in soybean and sunflower oils. The results in Table 3 show that XGBoost represents the highest *R* ^2^ scores and the lowest values for MAE and MSE across all cases. This exceptional performance can be attributed to several reasons that highlight the performance of the XGBoost model in capturing patterns in the data. First, XGBoost is an ensemble learning algorithm that combines multiple weak prediction models (decision trees) to create a robust model. This ensemble approach allows XGBoost to effectively capture complex relationships and interactions within the data, enabling accurate predictions of oil bleaching efficiency. Furthermore, XGBoost employs a gradient‐boosting technique that iteratively improves the model by minimizing the loss function.

**TABLE 3:** Regression models of ultrasound‐assisted bleaching of soybean and sunflower oil by machine learning and design expert methodology.

	Models	FNN	Random Forest	SVR	MT Lasso	Ridge	XGBoost	Gradient Boosting	RSM
CSU	MSE	3.557	1.981	10,224	7.144	9.209	3.370	1.850	ND
	MAE	1.132	0.820	1.694	1.620	1.610	0.906	0.810	ND
	*R* ^2^‐train	.830	.967	.800	.686	.806	.999	.980	.816
	*R* ^2^‐test	.760	.904	.808	.670	.738	.904	.880	.823
HUSU	MSE	1.711	0.978	34.334	15.189	16.727	1.664	1.552	ND
	MAE	0.784	0.550	2.756	2.557	2.708	0.664	0.587	ND
	*R* ^2^‐train	.906	.987	.784	.723	.784	.999	.972	.946
	*R* ^2^‐test	.900	.957	.777	.685	.770	.979	.962	.947
BUSU	MSE	2.455	1.0383	29.185	19.254	19.653	1.338	1.114	ND
	MAE	0.905	0.618	2.567	2.804	2.998	0.498	0.531	ND
	*R* ^2^‐train	.916	.994	.783	.681	.796	1	.996	.936
	*R* ^2^‐test	.901	.953	.736	.676	.706	.982	.981	.937
CSO	MSE	ND	63657.73	33.53	63653.62	60944.17	1.824	79572.67	ND
	MAE	ND	26.103	1.331	26.215	32.257	0.629	32.77	ND
	*R* ^2^‐train	ND	.917	.661	.431	.576	.999	.934	.602
	*R* ^2^‐test	ND	.686	.516	.565	.663	.902	.496	.624
HUSO	MSE	10.231	4.432	23.577	17.965	19.515	3.073	1.747	ND
	MAE	1.343	0.779	1.704	2.259	2.262	0.679	0.722	ND
	*R* ^2^‐train	.852	.983	.817	.733	.817	.999	.956	.972
	*R* ^2^‐test	.840	.962	.817	.743	.79	.911	.968	.974
BUSO	MSE	2.094	2.270	14.963	20.852	14.905	1.905	1.737	ND
	MAE	1.002	0.789	1.797	2.486	2.153	0.782	0.678	ND
	*R* ^2^‐train	.922	.995	.881	.838	.910	1	.996	.965
	*R* ^2^‐test	.915	.968	.864	.823	.857	.983	.985	.968

While comparing the results of ML regression models, it has been observed that the highest values of *R* ^2^‐train and *R* ^2^‐test, and subsequently the lowest values of errors, belong to XGBoost, followed by RF, and then GB. Based on the R^2^ and error values presented in Table 3, the ML models can be arranged from best to poorest performance as follows: XGBoost > Random Forest > Gradient Boosting > FNN > SVR > Ridge > MT Lasso.

**FIGURE 1:**
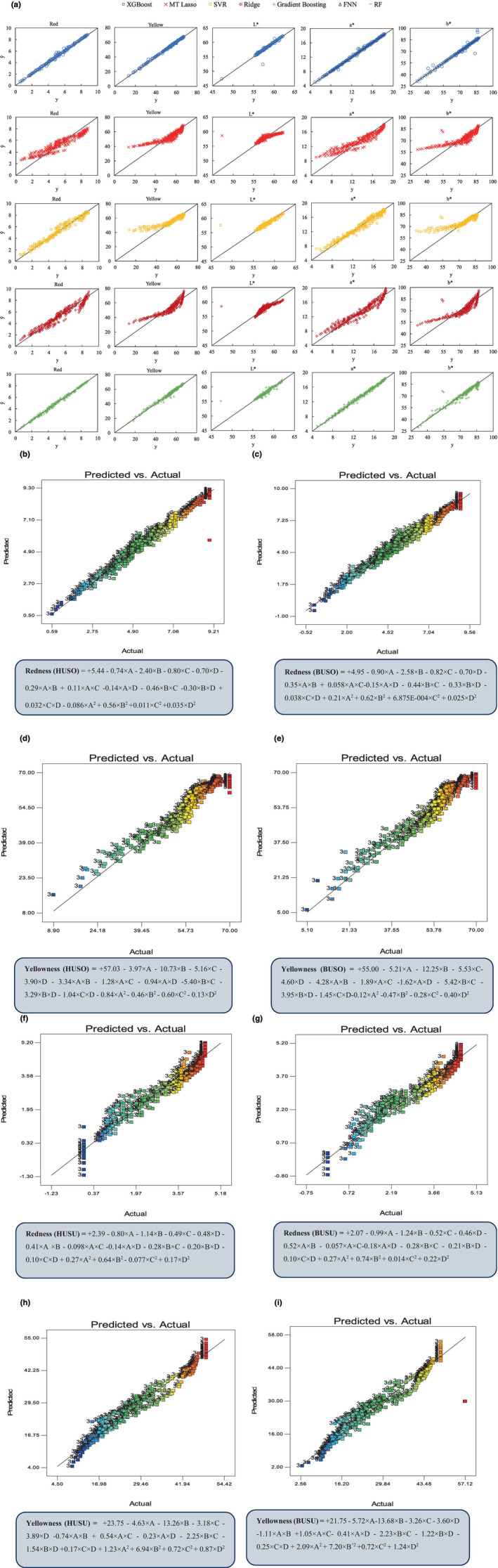
Results of different models of regression by ML (a) and RSM (b–i).

According to Table 3, the MT Lasso model exhibited poor performance compared to other models. The MT Lasso model utilizes a regularization technique that imposes a penalty on the model's coefficients, encouraging sparse solutions and feature selection. The poorer performance of the MT Lasso model could be ascribed to its specific regularization approach, which may not have been suitable for capturing the complexities and nuances of the pigment removal process in these cases. Moreover, to visualize and compare the performance of models, it is common practice to plot the actual targets against the predicted targets. This allows us to visually assess how well the models predict the target variable (Figure 1). To compare the soybean oil bleaching efficiency of control and UAB by all ML models, the scatter plot of the actual targets versus the predicted targets was calculated. The ideal condition would be a perfect fit, where the points lie along the line *y* = $\hat{y}$, where $\hat{y}$ represents the predicted value of the targets. As can be seen in Figure 1, the points for XGBoost are tightly clustered around the line *y* = $\hat{y}$, indicating a strong correlation between the predicted and actual targets. This implies that the XGBoost model achieves outstanding performance in predicting the target variables. The MT Lasso model's performance showed that this model deviates significantly from the ideal line *y* = $\hat{y}$, which indicates that the model might not be accurately predicting the target variable. This iterative process allows XGBoost to focus on the most important features and adjust the model's predictions accordingly, leading to superior performance.

As shown in Table 3, the results indicate that some models struggled to provide acceptable predictions, particularly for the control bleaching of soybean oil. In this regard, the FNN model, in particular, exhibited poor performance in capturing the patterns. The FNN's inability to capture the underlying data patterns might be attributed to issues such as model complexity, overfitting, or a lack of sufficient training data. Additionally, in this case, both the Random Forest and Gradient Boosting models displayed signs of overfitting. Overfitting occurs when a model becomes too complex and starts to memorize the training data instead of generalizing well to unseen data. Similarly, the study conducted by Deng et al. (2021) used XGBoost as a prominent model among practitioners in the field of ML to predict the minimum inhibitory concentration (MIC) of all non‐*typhoidal Salmonella*. It combines multiple decision trees, known as weak learners, to achieve enhanced performance through ensemble learning. This approach aims to improve the predictive capabilities of the algorithm by leveraging the strengths of individual decision trees and combining their results. Moreover, the XGBoost model demonstrated superior performance compared to other ML models in predicting MIC against *Salmonella enterica* (Nguyen et al., 2019) and *Klebsiella pneumoniae* (Nguyen et al., 2018). This was achieved independently of precompiled antimicrobial resistance (AMR) genes or polymorphisms. The XGBoost model exhibited higher accuracy and predictive capability in determining MIC values with a mean accuracy of 0.95, highlighting its efficacy in this specific context.

### 3.3 Comparison of ML and RSM results

By comparing the results obtained from RSM with the machine learning models used in this study, it is concluded that, on average, the performance of RSM was weaker than the XGBoost, Random Forest, and Gradient Boosting models and more successful than the other ML models. RSM and ML regression models serve different purposes and have distinct characteristics. RSM is primarily used for process optimization, relying on mathematical equations and assumptions about the relationship between input and response variables. It requires a limited number of observations and provides a simple equation for interpretation. On the other hand, ML regression models are employed for predicting outcomes based on inputs, without assuming any specific relationship. They can handle complex and non‐linear relationships, large datasets, and extrapolation. While RSM is simpler and more robust to noise, ML regression models offer flexibility and power in handling diverse scenarios.

### 3.4 Bayesian optimization and validation

Since the high consumption of bleaching clay in the bleaching oil process causes multiple problems, as mentioned in the introduction section, ultrasound was used as a combined technique to reduce bleaching clay. To minimize the amount of bleaching clay and energy consumption, the optimization process was conducted. To meet the requirement of a red color value below 2.5 during the oil bleaching process (Rossell, 1991), the optimization procedure focused on reducing the oil's red color. In the model developed in Python, Bayesian Optimization is used to efficiently search for optimal input parameters. Unlike traditional optimization methods that rely on gradient information or random search, Bayesian Optimization builds a probabilistic model (often a Gaussian process) to represent the function that needs to be optimized (Frazier, 2018). This model captures the uncertainty in the function's behavior. Instead of just looking at the local gradient and Hessian (curvature) information, Bayesian optimization uses all the information from the previous function evaluations to guide the search. By considering the uncertainty in the model, Bayesian Optimization can balance exploration (searching new regions of the parameter space) and exploitation (focusing on the most promising regions). This helps it find the optimal or near‐optimal solution with fewer function evaluations compared to other methods (Snoek et al., 2012). The experiments involved four factors: bleaching clay, time, power, and temperature. XGBoost was selected as the best regression model, and its results were subsequently inputted into the optimization model. Bayesian Optimization efficiently explores the parameter space to identify the combination of input parameters that minimizes the model output. For the optimization process, parameter bounds are defined to specify the range of values that each parameter can take. This ensures that the optimization process stays within reasonable and meaningful parameter values. Then the optimized samples were tested again to measure the accuracy of the XGBoost model. The optimal combination of input parameters is found by minimizing the model output.

To validate the optimization results, experiments were conducted using the optimal conditions obtained from the optimization process based on general factorials obtained by RSM and XGBoost Process Regression with ML (Table 4). The purpose of these experiments was to assess the effectiveness of the optimization method in achieving the desired outcomes. These results are compared to the predicted values obtained from the optimization method, and the error between the two sets of values is also expressed. This allows for a quantitative assessment of the accuracy of the optimization method in predicting the experimental outcomes.

**TABLE 4:** Red color for comparison between actual and predicted responses at optimum conditions [Ultrasound power (X1), clay content (X2), temperature (X3), and time (X4)].

	X1: 283 W, X2: 0.6%, X3: 58°C, X4: 19 min, HUSU	X1: 307 W, X2: 1.3%, X3: 45°C, X4: 23 min, HUSO	X1: 566 W, X2: 0.6%, X3: 20°C, X4: 58 min, BUSU	X1: 614 W, X2: 1.4%, X3: 46°C, X4: 25 min, BUSO
Experimental value	2.5	2.4	2.5	2.3
Predicted values by ML (XGBoost)	2.1	1.9	1.8	1.7
Error	0.4	0.5	0.7	0.6
	X1: 312 W, X2: 0.8%, X3: 53°C, X4: 23 min, HUSU	X1: 335 W, X2: 1.1%, X3: 63°C, X4: 17 min, HUSO	X1: 586 W, X2: 0.8%, X3: 21°C, X4: 52 min, BUSU	X1: 672 W, X2: 1.3%, X3: 52°C, X4: 26 min, BUSO
Experimental value	2.5	2.5	2.5	2.4
Predicted values by RSM	1.9	1.7	1.7	1.5
Error	0.6	0.8	0.8	0.9

where: Error = Experimentalresponse − Predictedresponse.

From Table 4, it was found that the error obtained by the ML was less than the RSM. The lower error between the predicted and experimental values suggests the superiority of the XGBoost model, and this model is more accurate in predicting the outcomes of the experiments.

### 3.5 Effect of ultrasonic power and modes on bleached oil color and oil pigments by ML

**FIGURE 2:**
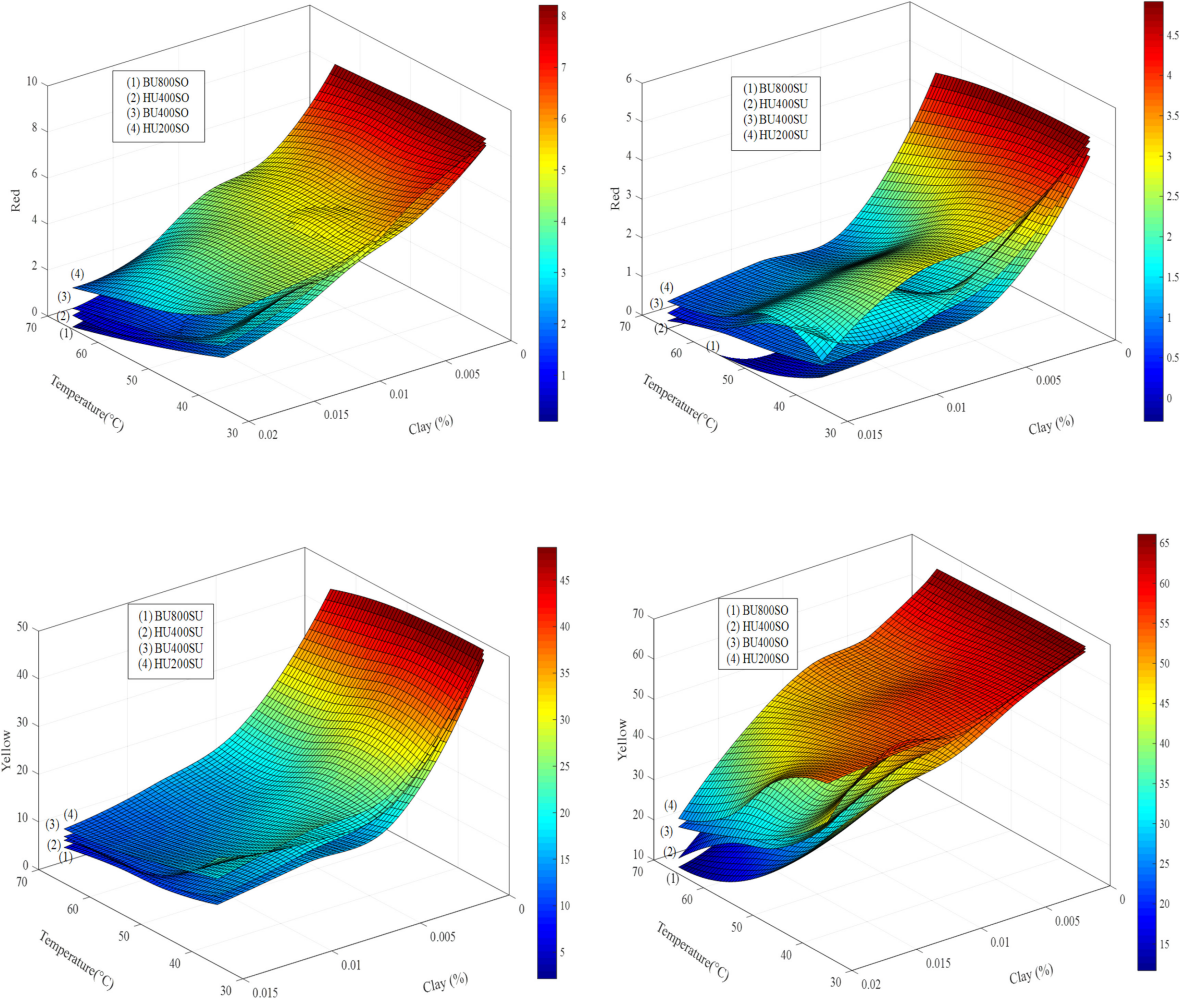
The content of red and yellow color after different bleaching conditions in HU400SO, BU400SO, HU400SU, BU400SU, HU800SO, BU800SO, HU800SU, and BU800SU.

**FIGURE 3:**
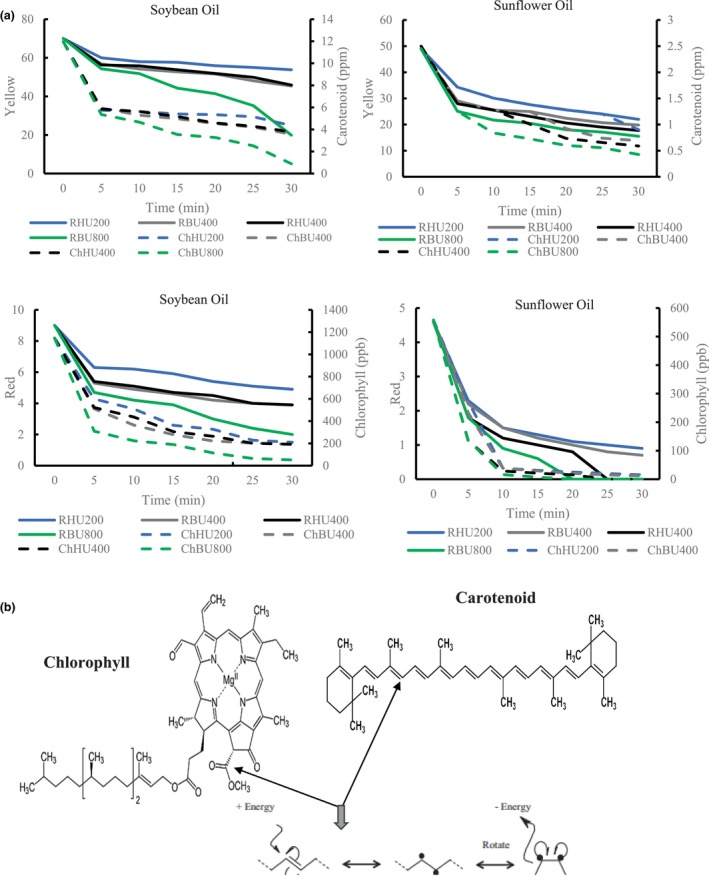
The correlation of the reduction of red color with chlorophyl removal and yellow color with carotenoid removal in HU400SO, BU400SO, HU400SU, BU400SU, HU800SO, BU800SO, HU800SU, and BU800SU (a). The structural changes of chlorophyll and carotenoid after ultrasonication (b).

**FIGURE 4:**
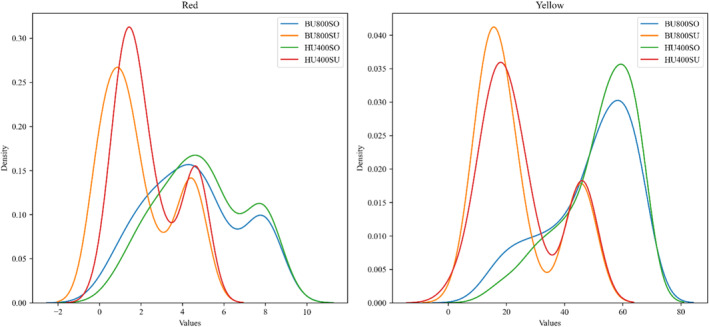
The density diagram visually displays the impact of different input features on each output parameter.

The strong effect of ultrasonic parameters on oil color was observed for all the investigated, as indicated by Figures 2, 3, 4. Significant and strong reductions in both red and yellow colors were observed for all vegetable oils tested when the temperature was increased from 35 to 65°C, power was increased from 200 to 400 W (for horn ultrasound) and 400 to 800 (for bath ultrasound), and time was increased from 5 to 30 min.

According to Jahouach‐Rabai et al. (2008), the collapse of bubbles and cavitation typically occurs when the oil is treated with ultrasound for a duration longer than 20 min. They also found that significant changes in the pigment and oil color were observed within a range of 20–30 min of ultrasound treatment. However, it is important to note that these changes were observed under the most severe conditions. The higher reduction rate in pigments observed during ultrasonic processing could be attributed to oxidation reactions facilitated by the interaction of free radicals formed during sonication, as suggested by Carail et al. (2015) and Tiwari et al. (2008). Additionally, pyrolysis occurring within the cavitation bubbles could contribute to the destruction of pigments (Tiwari et al., 2008). The generation of hydroxyl radicals and other free radicals during ultrasonic processing can damage and discolor chlorophylls and β‐carotene. This reaction is similar to the activity of the enzyme lipoxygenase, which produces hydroperoxide and free radical compounds that stimulate the bleaching of polyene compounds such as β‐carotene and chlorophyll. Therefore, the formation of free radicals and subsequent oxidative reactions induced by ultrasonic processing can lead to the degradation and discoloration of pigments in the oil (Figures 2, 3, 4).

### 3.6 Effect of bleaching clay concentration on bleached oil color and oil pigments by ML

**FIGURE 5:**
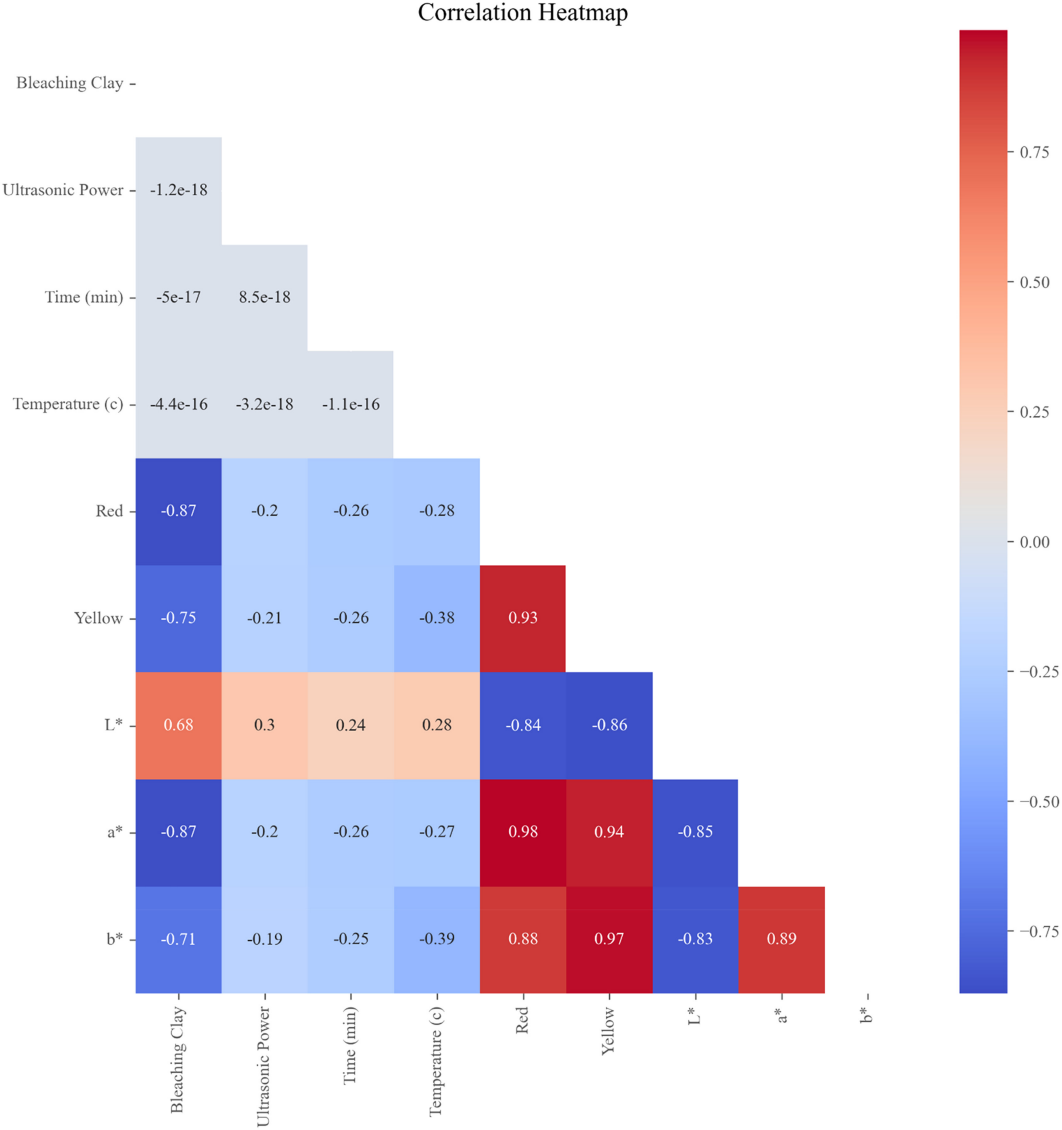
Correlation between input and output parameters in removing color in ultrasound‐assisted bleaching.

**FIGURE 6:**
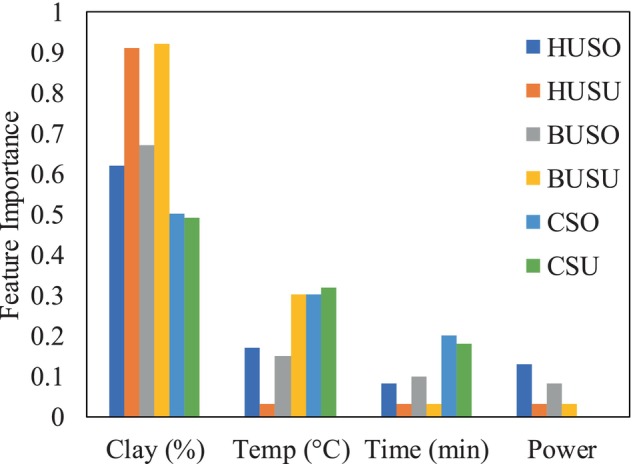
The importance of bleaching clay, temperature, time, and ultrasonic power for removing color using the XGBoost regression model.

As can be seen in Figure 5, bleaching clay shows a moderate negative correlation with the most color‐related output measurements (red, yellow, a*, b*), with correlation coefficients ranging from −0.71 to −0.87. This suggests that as the bleaching clay increases, the bleached color tends to decrease. On the other hand, ultrasonic power demonstrates a weak negative correlation with color, with correlation coefficients ranging from −0.19 to −0.21, indicating a slight decrease in color was observed when the ultrasonic power increased. The feature importance (bleaching clay, temperature, time, and ultrasonic power) of the XGBoost regression model is presented in Figure 6. The results showed that the importance of bleaching parameters were as follow; clay > temperature > ultrasonic power > time. In line with the results obtained by ML, based on the results from design expert methodology, the regression models for the effect of ultrasound power (*A*), clay content (*B*), temperature (*C*), and time (*D*) on oil color can be expressed as follows:

$$\text{Oil color}=\beta_{0}+\beta_{1}\times B+\beta_{2}\times C+\beta_{3}\times A+\beta_{4}\times D$$

where *β*_0_ is the intercept term; *β*_1_, *β*_2_, *β*_3_, and *β*_4_ are the coefficients for clay content, temperature, ultrasound power, and time, respectively.

Additionally, the combined application of clay and ultrasound power may have resulted in a synergistic effect on the reduction of pigment content and color. The process of sonication, carried out at different concentrations of solids, induces the release of energy through the collapse of cavitation bubbles (Abedi et al., 2015, 2020). The results indicate that the clay content has the most significant impact on oil color, followed by temperature, ultrasound power, and time. Based on the equation obtained by RSM, the combined influence of ultrasound power, clay content, temperature, and time has a more pronounced impact on the yellow color of the oil compared to the red color (Figure 1).

In addition to the use of clay, the application of ultrasound power in the process could have resulted in a synergistic effect on the reduction of pigment content and color. Sonication involves the use of high‐frequency sound waves to create and collapse tiny bubbles in a liquid, a phenomenon known as cavitation. When ultrasound is applied to a system containing solids, such as clay, the collapse of cavitation bubbles generates intense local energy. This energy release can facilitate the dispersion and interaction of the clay particles with the pigment molecules, leading to an enhanced reduction in pigment content and color. The synergistic effect of clay and ultrasound can be attributed to several factors. First, the cavitation bubbles created by ultrasound can physically disrupt the structure of the pigments, making them more accessible for interaction with the clay particles. Second, the energy released during bubble collapse can generate localized temperature and pressure changes, which can further enhance the breakdown of double bonds in the pigments, resulting in decolorization. Moreover, when cavitation occurs at the liquid–solid interface, energy release and bubble collapse effects can also induce high‐velocity particle‐to‐particle collisions, high shear forces, macro‐turbulence, and perturbations in microporous particles. These effects contribute to a surface cleaning effect, where contaminants or undesired substances on the surface are removed or reduced. The mechanical forces generated by the collapse of bubbles can promote the dispersion of clay particles, improving their contact with the pigments. As can be seen in Table 2, total pore volume increased, facilitating pigment adsorption on bleaching clay. Additionally, the increased mass transfer of oil pigments onto clay microporous surface particles occurs due to the enhanced mixing and dispersion of the pigments caused by cavitation. The high shear forces and macro‐turbulence created by cavitation promote the interaction and mixing of the pigments with the clay particles, leading to increased mass transfer and a more effective reduction in pigment content. A study conducted by Hu et al. (2023) stated that activated carbon effectively interacted with zearalenone via *π*–*π* interaction and adsorbed zearalenone during the bleaching process of corn oil. In another study, the adsorption of chlorophyll content of cold‐pressed hempseed oil using ultrasonication was reported by Aachary et al. (2016).

### 3.7 The effect of ultrasonic mode on bleaching efficiency by ML

**FIGURE 7:**
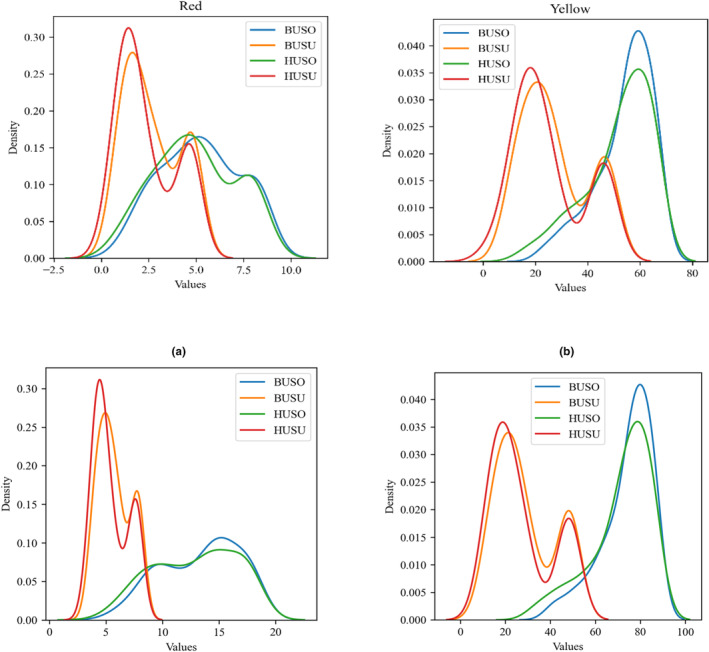
The density diagrams of HU400SO, BU400SO, HU400SU, BU400SU, HU800SO, BU800SO, HU800SU, and BU800SU after removing different colors.

The density diagrams showed the impact of ultrasonic mode (horn or bath) and power (200, 400, and 800 W) on the removal of colors (Figure 7). The results demonstrate that the impact of the ultrasonic probe is significantly greater than that of the ultrasonic bath, even when operated at the same power level. According to the density diagram at the same power, the effect of HU400SU and HU400SO on the reduction of red and yellow was significantly greater than that of BU400SU and BU400SO. The application of ultrasound through an ultrasonic bath can further mitigate the potential adverse effects of ultrasonic cavitation by maintaining mild reaction conditions. However, US baths have two main disadvantages that can significantly affect experimental repeatability and reproducibility. First, the distribution of US energy is not uniform, meaning only a small portion of the liquid volume near the US source experiences cavitation. Second, the power of the US bath decreases over time. In contrast, US probes focus their energy on a specific area of the sample, resulting in more efficient cavitation in the liquid. However, using US probes also has its limitations. Standing waves can form, causing the local intensity in a flask placed in a US cleaner to be highly sensitive to changes in experimental conditions, thereby affecting precision. The results of the study indicated that the use of an ultrasound bath was effective in reducing pigments and color in sunflower oil as compared to soybean oil.

### 3.8 The effect of bleaching temperature and time on bleaching efficiency by ML

Temperature (°C) and time (min) exhibit weak negative correlations ranging from −0.27 to −0.39 and −0.25 to −0.26 with bleached oil color. Additionally, a strong correlation was observed between redness and a* (0.98) as well as yellowness and b* (0.97). By increasing the intensity of the waves or extending the sonication time, more energy is imparted to the liquid, which can help overcome the cohesive forces and create the necessary conditions for cavitation to occur. The additional energy input can disrupt the cohesive forces, allowing the formation and collapse of cavitation bubbles. As the temperature of a liquid rises, the thermal energy increases the molecular motion and disrupts the intermolecular forces. This results in a reduction of the cohesive forces between the molecules, making the flow of the liquid easier and leading to a decrease in viscosity. The temperature range applied in the study can also affect the magnitude of the viscosity reduction. Higher temperature ranges generally lead to more pronounced changes in viscosity as the thermal energy becomes more significant in disrupting intermolecular forces. Mohammadian et al. (2013) found that ultrasonic radiation can generate heat within a liquid, leading to a reduction in density, surface tension, and viscosity. The generation of heat through ultrasonic radiation can increase the temperature of the liquid, which in turn affects its physical properties. The reduction in viscosity due to temperature changes is influenced by several factors. These may include the molecular structure and composition of the liquid, the strength of intermolecular forces, and the presence of additives or impurities.

### 3.9 The effect of oil composition on bleaching efficiency by ML

The order of the sonication effect on color reduction in bleached soybean oil was greater than that in sunflower oil. The results were consistent with Abedi et al. (2020) that oil bleaching efficiency was related to the type of oil (oil fatty acids and oil viscosity). It is important to note that the viscosity of a liquid affects its resistance to flow and the strength of the cohesive forces between its molecules. Therefore, more viscous liquids will generally require more intense or prolonged sonication to achieve cavitation compared to less viscous liquids (Hamida & Babadagli, 2007; Hamidi et al., 2014; Mohammadian et al., 2013).

The variation in viscosity among edible oils can be influenced by factors such as temperature and fatty acid composition. The studies by Santos et al. (2005), Fasina and Colley (2008), and Abedi et al. (2020) likely provide further insights into how the temperature and fatty acid composition of edible oils impact their viscosity. Temperature plays a significant role in determining the viscosity of oils (Bouchelkia et al., 2023). The reduction in viscosity due to temperature changes is influenced by factors such as ultrasonic cavitation and boundary friction. When fluids are exposed to ultrasonic waves, the phenomenon of ultrasonic cavitation generates heat, which can contribute to a reduction in viscosity. The viscosity of the oils decreased by approximately 30% for every 10°C increase in temperature. The higher thermal movement among molecules at higher temperatures reduces intermolecular forces, making flow among the molecules easier and resulting in a decrease in viscosity. However, it is important to note that the increase in viscosity of the oil can enhance the cavitation threshold, meaning that higher viscosity oils may require more intense ultrasonic treatment to induce cavitation and achieve viscosity reduction. Different types and proportions of fatty acids present in oils can influence their molecular structure and packing, which in turn affects viscosity. For example, oils with a higher proportion of saturated fatty acids tend to have higher viscosities compared to oils with higher proportions of unsaturated fatty acids. The concentration of polyunsaturated chains in edible oils has a stronger influence on viscosity compared to monounsaturated and saturated chains. This is because polyunsaturated chains contain more *π* bonds, which result in stronger intermolecular interactions between the *π* electrons of the double bonds. These stronger interactions can increase the viscosity of the oil. On the other hand, a more extended chain structure, which is often associated with polyunsaturated chains, can make flow easier and reduce viscosity. This is because the extended chain allows for more freedom of movement, resulting in lower resistance to flow. The occurrence of cavitation in a liquid medium requires that the negative pressure in the rarefaction area of the wave function surpasses the cohesive forces within the liquid. Consequently, cavitation is more challenging to form in viscous liquids where the cohesive forces are stronger and more vigorous. In viscous liquids, the cohesive forces between molecules are more pronounced, making it harder for the negative pressure to overcome them and initiate cavitation. As a result, higher intensity waves or longer sonication times are typically required to induce cavitation in viscous liquids. The study by Mercantili et al. (2015) supports the idea of viscosity reduction in oils after sonication. They observed that the viscosity of bleached oils decreased with increasing power, temperature, and sonication time. The reduction in viscosity can be attributed to various factors, including the collapse of cavitation bubbles, heat production, microjetting, and the presence of high local velocities of liquid layers. These phenomena generate local shear forces that disrupt adhesion forces between molecules, leading to a breakdown in viscosity. Additionally, during the collapse of cavitation bubbles, water molecules can be partially degraded into°OH and°H radicals. These radicals can decompose attractive forces among macromolecules, further contributing to the reduction in viscosity (Hamidi et al., 2014). Poesio et al. (2002) proposed that a reduction in the pressure gradient of a fluid exposed to ultrasonic waves can also cause a decrease in fluid viscosity. This suggests that the application of ultrasonic waves can directly affect the fluid's viscosity by altering the pressure gradients within the system.

## 4 CONCLUSION

The results highlight the strengths of XGBoost in accurately predicting the color removal process for both soy and sunflower oils. This indicates that the optimization based on the XGBoost model is more effective in achieving the desired results compared to other methods (RSM). The regression analysis of soybean oil and sunflower oil using two modes of ultrasonic treatment, namely bath and horn, revealed that bath ultrasonic is more effective in reducing color for oils with low color intensity, such as sunflower oil. On the other hand, horn ultrasonic treatment is recommended for soybean oil, which contains high levels of chlorophyll pigments and requires a more targeted approach for color removal. These results highlight the importance of considering the specific characteristics of different oils when selecting the appropriate ultrasonic treatment method for color reduction. Based on our findings, the implementation of the ultrasonic process has the potential to diminish the consumption of clay, minimize the duration and temperature required for the bleaching process, and conserve energy while achieving higher throughput.

## AUTHOR CONTRIBUTIONS

**Elahe Abedi:** Conceptualization (equal); methodology (equal); project administration (equal); resources (equal); writing – original draft (equal); writing – review and editing (equal). **Mehran Sayadi:** Investigation (equal); methodology (equal); validation (equal); visualization (equal). **Maryam Mousavifard:** Software (equal); supervision (equal); validation (equal). **Farzad Roshanzamir:** Project administration (equal); software (equal); validation (equal).

## CONFLICT OF INTEREST STATEMENT

The authors declare that they have no conflict of interest.

## ETHICS STATEMENT

This article does not contain any studies with human participants or animals performed by any of the authors.

## CONSENT TO PARTICIPATE

The present paper has been approved by all the named authors.

## CONSENT FOR PUBLICATION

The present paper, which is original, has not been published before and is not currently being considered for publication elsewhere.

## Supporting information


Appendix S1

## Data Availability

Research data are not shared.
