# Supplementary material for: A comparative study on bath and horn ultrasound‐assisted modification of bentonite and their effects on the bleaching efficiency of soybean and sunflower oil: Machine learning as a new approach for mathematical modeling
Source: Food Sci Nutr. 2024 Jul 9;12(9):6752–71. doi: 10.1002/fsn3.4300 (PMC11561808; doi:10.1002/fsn3.4300)
Supplement: Supplementary file 1 — Appendix S1 [file FSN3-12-6752-s001.docx]

**Supplementary file**

# Machine Learning Regression Models

**1. Feedforward Neural Network (FNN)**

An FNN, or feedforward neural network, is a type of artificial neural network which comprises of an input layer, one or more hidden layers, and an output layer. The purpose of an FNN is to process data in a forward direction, starting from the input layer, traversing through the hidden layers, and ultimately reaching the output layer. This enables the network to make predictions or decisions based on the input data. (Sazli, 2006).

The neurons in the hidden layers receive a weighted sum of the outputs from the neurons in the previous layer. To introduce non-linearity and help the model learn complex patterns in the data, the weighted sum is passed through an activation function. Finally, the output layer produces the final predictions or outputs of the model. (Ben Braiek and Khomh, 2023).

The model used in this research consists of three hidden layers, each with a ReLU (Rectified Linear Unit) activation function. The first hidden layer has 128 neurons, the second hidden layer has 128 neurons, and the third hidden layer has 64 neurons.

The activation function is a crucial component of an FNN. ReLU is often used in hidden layers due to its simplicity, computational efficiency and ability to handle the vanishing gradient problem.

During the training process, the FNN learns to adjust the weights and biases of the neurons to minimize the difference between the predicted outputs and the true outputs. This is done by iteratively updating the model's parameters using an optimization algorithm, such as gradient descent, and a loss function that measures the difference between the predicted and true outputs (the loss function is set to mean squared error). The optimization algorithm adjusts the weights and biases in a way that minimizes the loss function.

A simple feedforward neural network regression model can be represented as follows (Wu et al., 2023):

y = f(W_2_ × f(W_1_ × X + b_1_) + b_2_)

where y represents the predicted output or target variable, and X represents the input features or independent variables. The weights W_1_ and W_2_ correspond to the neural network's hidden and output layers, respectively. The biases b_1_ and b_2_ are associated with the hidden and output layers, respectively.

**2. Random Forest Regressor**

Random forest is a popular ensemble learning method that can be useful for a variety of regression applications (Mateo Pérez et al., 2021; Zhou et al., 2019). To prevent overfitting of the training set, the algorithm generates multiple decision trees using bootstrapping and averages the results of each tree. By tuning hyperparameters like the number of decision trees, we can enhance the quality of the random forest algorithm.

In the prediction phase, each decision tree in the Random Forest Regressor predicts the target variables for a given input independently. In the case of multi-output regression, each decision tree predicts all the target variables simultaneously. Finally, the prediction for each target variable is obtained by averaging or taking the median of the predictions from all the decision trees in the ensemble. This operation of averaging or taking the median helps to reduce the variance and improve the overall accuracy of the prediction (Breskvar et al., 2018).

Each decision tree in the Random Forest ensemble method is capable of predicting multiple continuous target variables simultaneously. The splitting criterion in each tree is adjusted to consider the correlation or relationship between the target variables. During training, the trees learn to capture the dependencies and interactions between the input features and the multiple output variables.

y = Σ (h_i_(X) × w_i_)

In this equation h_i_(X) represents the prediction made by each individual decision tree in the random forest. w_i_ represents the weight or importance assigned to each decision tree's prediction (Breiman, 2001).

**3. Support Vector Regression (SVR)**

Support vector regression (SVR) is an extension to the Support Vector Machine (SVM) classification algorithm and is used to handle regression problems in supervised machine learning (Zhang and O’Donnell, 2020). Although originally designed for single-output regression, SVR can also handle multi-output regression tasks by using a kernel trick to transform input data into a higher-dimensional feature space (Borchani et al., 2015; Li et al., 2020). In this code the Radial Basis Function (RBF) kernel is used which allows SVR to capture complex relationships between the input features and the target variables. During the training phase, SVR finds a hyperplane in the transformed feature space that best fits the data points while satisfying a certain margin of error (Zhang and O’Donnell, 2020). SVR aims to minimize the error between the predicted outputs and the true outputs while maximizing the margin around the hyperplane. The margin is controlled by two hyperparameters: C, which determines the penalty for data points outside the margin, and epsilon (ε), which defines the width of the margin. To handle multi-output regression tasks, SVR can be extended using different approaches. In this research Joint Models are used. In this method a single SVR model is trained that predicts all the output variables simultaneously. This can be done by incorporating the relationships between the output variables into the model formulation (Jana et al., 2022).

y = Σ (α_i_ × K(X_i_, X)) + b

In this equation, α_i_ represents the Lagrange multipliers associated with the support vectors, K(X_i_, X) represents the kernel function applied to the support vectors and the input features, and b represents the bias term.

**4.** **Multi-Task Lasso**

Multiple tasks can refer to learning multiple output targets using one input source, learning a single output target using multiple input sources, or a combination of both. The objective functions of multitask learning (MTL) can vary depending on the definition of multiple tasks. In cases where the original data representation is high dimensional and there are limited examples to solve a regression or classification problem, learning algorithms without prior knowledge will perform poorly due to a lack of data to estimate model parameters reliably. MTL, as a type of machine learning, aims to solve multiple tasks simultaneously by leveraging information from related tasks to address the scarcity of data (Thung and Wee, 2018). Multi-Task Lasso extends the Lasso regression algorithm to handle multiple outputs. Lasso regression is a linear regression model (Cui et al., 2018) that includes a regularization term to prevent overfitting and encourage sparsity in the model. The Lasso regression minimizes the sum of squared errors between the predicted values and the true values, subject to the constraint that the sum of the absolute values of the coefficients is less than or equal to a constant multiplied by the number of features. In Multi-Task Lasso, each target variable is modeled as a linear combination of the input features with its own set of coefficients. The objective function of Multi-Task Lasso consists of two terms: the sum of squared errors between the predicted values and the true values, and the sum of the absolute values of the coefficients across all output variables. The regularization term encourages sparsity not only within each target variable but also across all the target variables simultaneously. The Lasso model can be formulated as follows (Emmert-Streib and Dehmer, 2019)

$$\arg min\left\{ \frac{1}{(2 \times n)} \times{||Y - X\beta||}_{2}^{2} + \lambda\times\sum_{i=1}^{p} w_{j}\left| \beta_{j} \right| \right\}$$

In this equation β is the matrix of coefficients, where each column represents the coefficients for a different target variable. n is the number of samples in the dataset, p is the number of predictors. λ is the tuning or regularization parameter.

**5. Ridge regression**

ridge regression was proposed as a method to enhance the reliability of multiple linear regression estimates by Hoerl and Kennard, 1970. It does so by decreasing the standard error and introducing a degree of bias. Ridge regression is particularly useful in preventing multicollinearity by shrinking parameters and reducing model complexity through coefficient shrinkage. The prediction of multiple linear regression can be done using the following equation, as stated by Devi et al. (2023):

y = Xβ + ε

In this equation, the dependent variable is represented by the column vector y with dimensions n× 1. The explanatory variables are observed in the matrix X with dimensions n× p, where p is the number of variables and p ⩽ n. The matrix X has a full rank of p. The regression coefficients are denoted by the column vector β, which has dimensions p ×1 and is unknown. The vector ε represents the random errors and has dimensions n× 1.

In ridge regression model *β* is changed from (*X^T^X*)^-1^*X^T^Y* multiple linear regression to (*X^T^X* + *kI*)^-1^*X^T^Y* by adding a penalty term k (ridge parameter) (Devi et al., 2023).

In this paper, the Ridge Regression Python code uses Grid Search CV from scikit-learn for hyperparameter tuning. The best hyperparameter alpha value is found 1. Both Ridge regression and Multi-Task Lasso models use L2 regularization to prevent overfitting and improve generalization performance (Pereira et al., 2016). This regularization term penalizes large coefficients and encourages smaller and more balanced coefficients.

**6. XGBoost (Extreme Gradient Boosting)**

XGBoost is a highly efficient and scalable machine learning algorithm used for regression tasks. It belongs to the gradient boosting ensemble method (Chen and Guestrin, 2016), which combines multiple weak learners (decision trees) to create a strong predictive model. As for the base learners, XGBoost employs decision trees. These trees are constructed in a greedy manner, finding the best feature and threshold to minimize the loss function (Sagi and Rokach, 2021). The model's complexity and overfitting can be controlled by adjusting the tree depth and other hyperparameters.

To prevent overfitting and improve generalization, XGBoost incorporates regularization techniques (Chen and Guestrin, 2016). The strength of regularization is controlled by parameters like alpha and lambda. Feature importance estimation is another useful feature of XGBoost. It calculates the relative importance of each feature based on the number of times it is used for splitting across all trees in the ensemble (Alsahaf et al., 2022). This information aids in feature selection and understanding variable impact. XGBoost is designed to be highly scalable and efficient, making it suitable for large datasets.

**7. Gradient Boosting Regressor**

The Gradient Boosting Regressor is an ensemble machine learning algorithm that combines multiple decision trees to build a strong predictive model for regression tasks, belonging to the gradient boosting method similar to XGBoost (Sipper and Moore, 2022). The base learners in Gradient Boosting Regressor are decision trees which are constructed in a greedy manner, finding the best feature and threshold to minimize the loss function. The model's complexity and overfitting can be controlled by adjusting the hyperparameters (Sipper and Moore, 2022).

Gradient Boosting Regressor and XGBoost share several similarities due to their common foundation in gradient boosting. Both methods use gradient boosting to optimize the model's performance and combine decision trees as base learners. They involve an iterative process of sequentially adding weak learners to improve predictions.

**References**

Alsahaf, A., Petkov, N., Shenoy, V., Azzopardi, G., 2022. A framework for feature selection through boosting. Expert Systems with Applications 187, 115895.

Ben Braiek, H., Khomh, F., 2023. Testing feedforward neural networks training programs. ACM Transactions on Software Engineering and Methodology 32, 1–61.

Borchani, H., Varando, G., Bielza, C., Larranaga, P., 2015. A survey on multi‐output regression. Wiley Interdisciplinary Reviews: Data Mining and Knowledge Discovery 5, 216–233.

Breiman, L., 2001. Random Forests. Machine Learning 45, 5–32. https://doi.org/10.1023/A:1010933404324

Breskvar, M., Kocev, D., Džeroski, S., 2018. Ensembles for multi-target regression with random output selections. Machine Learning 107, 1673–1709.

Chen, T., Guestrin, C., 2016. Xgboost: A scalable tree boosting system. Presented at the Proceedings of the 22nd acm sigkdd international conference on knowledge discovery and data mining, pp. 785–794.

Cui, L., Xie, X., Shen, Z., Lu, R., Wang, H., 2018. Prediction of the healthcare resource utilization using multi-output regression models. IISE Transactions on Healthcare Systems Engineering 8, 291–302.

Devi, M., Malik, D., Mehala, V., Mishra, P., 2023. Measuring Variability and Factors Affecting the Agricultural Production: A Ridge Regression Approach. Annals of Data Science 1–14.

Emmert-Streib, F., Dehmer, M., 2019. High-dimensional LASSO-based computational regression models: regularization, shrinkage, and selection. Machine Learning and Knowledge Extraction 1, 359–383.

Hoerl, A.E., Kennard, R.W., 1970. Ridge regression: Biased estimation for nonorthogonal problems. Technometrics 12, 55–67.

Jana, D.K., Bhunia, P., Adhikary, S.D., Bej, B., 2022. Optimization of effluents using artificial neural network and support vector regression in detergent industrial wastewater treatment. Cleaner Chemical Engineering 3, 100039.

Li, Y., Sun, H., Yan, W., Zhang, X., 2020. Multi-output parameter-insensitive kernel twin SVR model. Neural Networks 121, 276–293.

Mateo Pérez, V., Mesa Fernández, J.M., Villanueva Balsera, J., Alonso Álvarez, C., 2021. A Random forest model for the prediction of FOG content in inlet wastewater from urban WWTPs. Water 13, 1237.

Pereira, J.M., Basto, M., Da Silva, A.F., 2016. The logistic lasso and ridge regression in predicting corporate failure. Procedia Economics and Finance 39, 634–641.

Sagi, O., Rokach, L., 2021. Approximating XGBoost with an interpretable decision tree. Information Sciences 572, 522–542.

Sazli, M.H., 2006. A brief review of feed-forward neural networks. Communications Faculty of Sciences University of Ankara Series A2-A3 Physical Sciences and Engineering 50.

Sipper, M., Moore, J.H., 2022. AddGBoost: A gradient boosting-style algorithm based on strong learners. Machine Learning with Applications 7, 100243.

Thung, K.-H., Wee, C.-Y., 2018. A brief review on multi-task learning. Multimedia Tools and Applications 77, 29705–29725.

Wu, Y., Cheng, S., Li, Y., Lv, R., Min, F., 2023. Sequential three-way decisions with a single hidden layer feedforward neural network. arXiv preprint arXiv:2303.07589.

Zhang, F., O’Donnell, L.J., 2020. Support vector regression, in: Machine Learning. Elsevier, pp. 123–140.
